# Physical and Mechanical Behaviour of Sugarcane Bagasse Fibre-Reinforced Epoxy Bio-Composites

**DOI:** 10.3390/ma13235387

**Published:** 2020-11-27

**Authors:** Lalta Prasad, Shiv Kumar, Raj Vardhan Patel, Anshul Yadav, Virendra Kumar, Jerzy Winczek

**Affiliations:** 1 Department of Mechanical Engineering, National Institute of Technology Uttarakhand, Srinagar 246174, India; laltaprasad@nituk.ac.in; 2 Department of Mechanical Engineering, GB Pant Institute of Engineering and Technology, Garhwal 246194, India; shivkumar23@gmail.com; 3 Department of Mechanical Engineering, Sherwood College of Engineering Research and Technology, Barabanki 225001, India; rvpatel.knit@gmail.com; 4 Membrane Science and Separation Technology Division, CSIR-Central Salt and Marine Chemicals Research Institute, Bhavnagar 364002, India; anshuly@csmcri.res.in; 5 Department of Mechanical Engineering, Kamla Nehru Institute of Technology, Sultanpur 228118, India; veer.iitdmech@gmail.com; 6 Faculty of Mechanical Engineering and Computer Science, Częstochowa University of Technology, 42-201 Częstochowa, Poland

**Keywords:** natural fibre, reinforcement, sugarcane bagasse, polyester resin, bio-composite

## Abstract

In this study, experiments are performed to study the physical and mechanical behaviour of chemically-treated sugarcane bagasse fibre-reinforced epoxy composite. The effect of alkali treatment, fibre varieties, and fibre lengths on physical and mechanical properties of the composites is studied. To study the morphology of the fractured composites, scanning electron microscopy is performed over fractured composite surfaces. The study found that the variety and lengths of fibres significantly influence the physical and mechanical properties of the sugarcane bagasse-reinforced composites. From the wear study, it is found that the composite fabricated from smaller fibre lengths show low wear. The chemically-treated bagasse-reinforced composites fabricated in this study show good physical and mechanical properties and are, therefore, proposed for use in applications in place of conventional natural fibres.

## 1. Introduction

The abundant agricultural/industrial waste generated from modern technologies has proved to be a barrier to sustainable development. The natural fibre-reinforced composites have been identified as a potential substitute in various applications due to their availability, cost-effectiveness, non-toxicity, and biodegradability [1]. Moreover, the natural fibre-reinforced composites display excellent properties such as high strength and stiffness that make them an excellent alternative to glass or carbon fibres for high strength applications such as construction [1,2]. Various natural fibres have been reported to be used for the fabrication of composites such as Jute, Coir, Sisal, Pineapple, Ramie, Bamboo, Banana, Hemp, Bagasse, Coconut, Flax, and Curaua [2,3,4,5,6,7]. The hybridising of composites improves the tensile strength and modulus of curaua/glass fibre composites [7].

Bagasse is the dry pulpy residue left out after the extraction of sugarcane. Apart from sugarcane, other plants also yield bagasse such as cassava, agave, and guayule [8,9]. A study of the layering pattern of hybrid composites of epoxy novolac reinforced with short bagasse and coir fibres stated that the tri-layer’s tensile properties are better compared to bi-layer composites [10]. The morphology of uncarbonised and carbonised composites of different bagasse particles show that the microstructure of the polymer composites’ is essential and responsible for the increase/decrease in mechanical properties [11]. The morphology studies on bagasse-reinforced composites by scanning electron microscopy (SEM) revealed that three layers of sisal fibres and the core layer of bagasse fibres produce excellent mechanical properties [12,13]. Omrani et al. [14] analysed the tensile properties of flax fibre yarns, fabrics, and composites. The study revealed the effect of the weaving process on tensile behaviour of un-impregnated fabrics. A parametric study on bagasse fibre-reinforced vinyl ester discovered that the fibre content influences the tensile and flexural strength [15]. The impact tests conducted on the specimen of the polyester matrix composite reinforced with different contents of bagasse fibre showed that the absorption capacity of impact increases with the increase in the content of bagasse fibre in the composite [16].

Chemically-treated natural fibre (in particular bagasse)-reinforced composites have been studied extensively. The chemical treatment of the raw fibres improves the interaction between the fibre and the epoxy matrix. The alkaline pre-treated and KMnO_4_ treated fibres improve the mechanical and thermal properties of composites [17,18]. A comparative study on untreated Bagasse composites with treated fibres with 10% NaOH revealed that the hardness of the treated composites decreased while their tensile strength increased [19]. The chemical treatment (1% NaOH and 1% acrylic acid) enhances the bonding strength between fibre and polymer and reduces water absorption of the composites [20].

It is expected that the improved properties of bagasse-reinforced composites may gain attention in sustainable development owing to their advantages with regard to ecological concern. The bagasse fibres are readily available as agricultural waste. The waste can be turned into a source of bio-composite that has many applications and an edge over conventional composites. This study’s main objective is the effective utilisation of bagasse and fabrication of a new class of epoxy-based composites reinforced with different lengths and varieties of sugarcane bagasse fibre. The bagasse fibre used to reinforce the composites was treated using NaOH solution. We also evaluated the physical and mechanical behaviour of the fabricated composites using hardness, tensile, wear, water absorption, and impact resistance tests.

## 2. Materials and Methods

### 2.1. Materials

In the present study, four varieties of sugarcane fibre were used (Cos 8436, CoJ 88, CoS 767, and Co 0293) as they are readily available in Uttarakhand, India (North West Zone). The epoxy resin LY 556 (matrix material) and HY 951 (epoxy hardener) were used in the present work and procured from the Northern Polymers, New Delhi, India. The properties of the sugarcane varieties are given in Table 1. The mechanical separation was used for separating the fibres. The fibres of different lengths (5, 10, and 15 mm) were cut using scissors. All the fibres were kept in 5 % aqueous NaOH solution for 24 h for the chemical treatment. 

### 2.2. Composite Fabrication

A conventional hand layup technique was used to fabricate the composite slab. The hardener and low temperature curing epoxy resin were mixed in a ratio of 1:9 by weight. The fibres were added to the epoxy in the ratio of 3:10 by weight. A mould of dimensions 220 × 220 × 20 mm^3^ made of plywood was used for composite fabrication. The mylar film was spread on the pattern first, and a releasing agent (silicon-free spray) was sprayed over mylar film to ensure the safe removal of composite from the mould. A constant load of 25 kg was applied to ensure the proper curing of the composites at room temperature for 24 h. The weight percent of sugarcane bagasse fibre and epoxy resin in each composite was fixed. The air bubbles which were entrapped during fabrication were removed by sliding rollers. Specimens were cut from the composites with dimensions as per the ASTM standard (physical, mechanical, and wear tests). The composition and designation of the composites used in the present study are shown in Table 2. 

### 2.3. Characterisation of Composite

#### 2.3.1. Density Test

The simple water absorption technique was used to determine the actual density of the fabricated composites. The theoretical density of composite materials was determined by the method given in our previous work [21].

#### 2.3.2. Water Absorption Test

The water absorption studies of the composites were performed as per the standard of ASTM D 570-98 [22]. The weights of the samples were taken before and after dipping them to regular water for 24 h. The samples were cleaned after removing from water and weighed. The weight of the test samples was measured regularly at the intervals of 24 h—i.e., at 24, 48, 72, 96, and 120 h. The weight difference of the samples shows the measure of the moisture content of the samples.

#### 2.3.3. Wear Test

ASTM G99 standards [23] have been used to calculate the wear of the samples. The pin-on-disc apparatus (Ducom, Bangalore, India) was used to determine the wear of composites during sliding. This test was conducted using a cylindrical specimen of diameter 8 mm and height 35 mm. Wear track diameter of 60 mm was taken. A 25 N load and the 800 RPM speed was kept for 10 min for each composite. The wear of particular samples was the measure of the difference between the initial and final length.

#### 2.3.4. Tensile Strength and Elongation at Breakpoint Test

The tensile test of the composite sample was carried out as per the ASTM D638-02A standards [24]. The specific points of interest are the point where the peak stress occurs in the testing, also called the ultimate tensile strength (UTS). The mean values of three identical test data were reported for each specimen. The constant test grip-clamp distance of 50 mm and the crosshead speed of 2 mm s^−1^ were taken for all test samples.

#### 2.3.5. Impact Strength Test

The impact test was performed to understand the toughness of the composites using a Veekay instrument (Model-I91, Chennai, India). The Charpy impact test was carried as per ASTM E23 standard [25] to measure the impact behaviour of the composites. The standard sample size (55 × 10 × 10 mm^3^) was taken, and a V-notch was made at an angle of 45° with a root depth of 2 mm.

#### 2.3.6. Hardness Test

The computerised Vickers hardness tester (Model: VM50PC, Kolhapur, India) machine with load accuracy ± 1% of the nominal load value was used to determine the hardness of the composites. For making the sharp indentations on the specimens, a rigid precision diamond indenter (136° pyramid) was used in this test with a 5 kgf load.

## 3. Results and Discussions

### 3.1. Physical Behaviour of Composites

**Figure 1:**
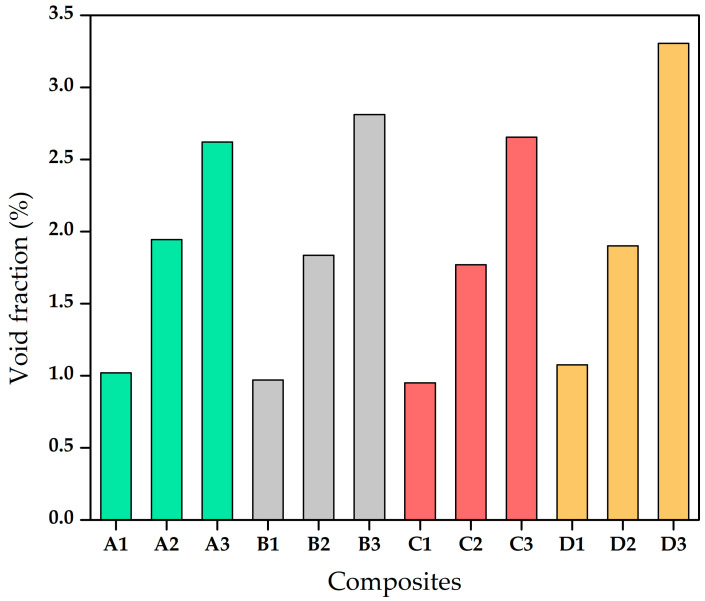
Void fraction (%) of composites.

The existence of void content significantly affects the physical and mechanical properties of the composites. The theoretical and experimental densities and the corresponding void contents for the composites are tabulated in Table 3. Although maximum possible measures were taken during the fabrication of composites to avoid the voids, the voids cannot be avoided in the hand layup technique. Therefore, it is essential to determine the void content of the composites and its effects on the properties of the composites. The voids are responsible for the density difference in theoretical and experimental. Figure 1 depicts the volume fraction of void of the composites. The percentage of voids was found to be the highest in D variety with 15 mm fibre length. The 5 mm length of bagasse fibre-reinforced epoxy composite showed the least void content as the short fibre lengths led to compact composites. The coarse surface of the natural fibre after treatment led to a reduction in hydroxyl groups which implies more contact space available for cross-linking between the fibre and the matrix material. This resulted in a higher density of the chemically treated bagasse fibre-reinforced composites [26].

**Figure 2:**
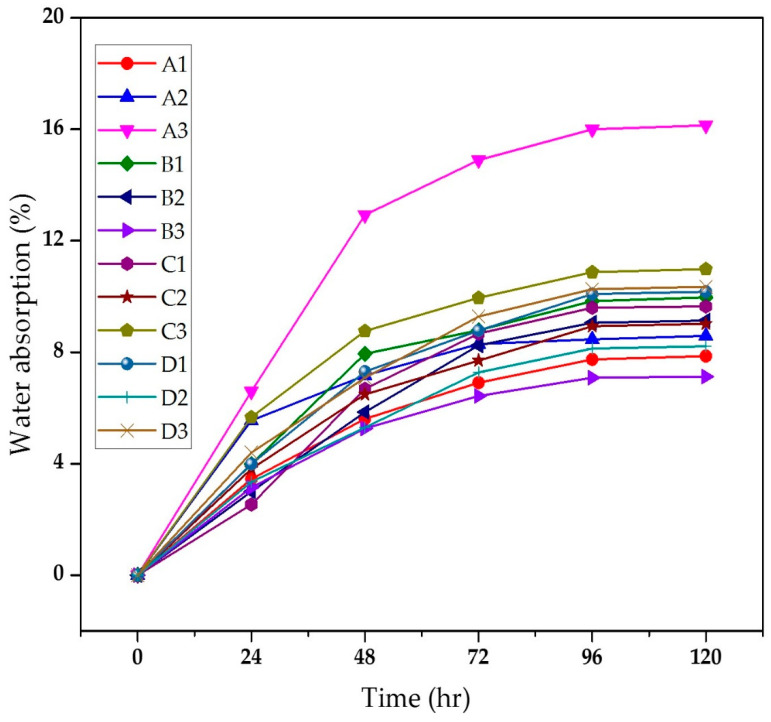
Water absorption (%) of composites.

Figure 2 shows the water absorption behaviour of the composites. It can be seen that for all the composites, a steady-state condition was achieved after 96 h of immersion. Similar trends for water absorption have been reported in the literature [27]. Due to the chemical treatment of bagasse fibres, the interaction of the fibres with matrix material increased leading to a compact composite [26]. Hence, a minimal amount of water was diffused through the material. The chemically-treated composites showed excellent water absorption resistance due to the reduction in hydroxyl groups in the fibre cell wall after chemical treatment [28].

### 3.2. Mechanical Behaviour of Composites

The mechanical properties of the different varieties and lengths of bagasse fibre-reinforced epoxy composite under constant fibre loading were investigated and are presented in Table 4. 

**Figure 3:**
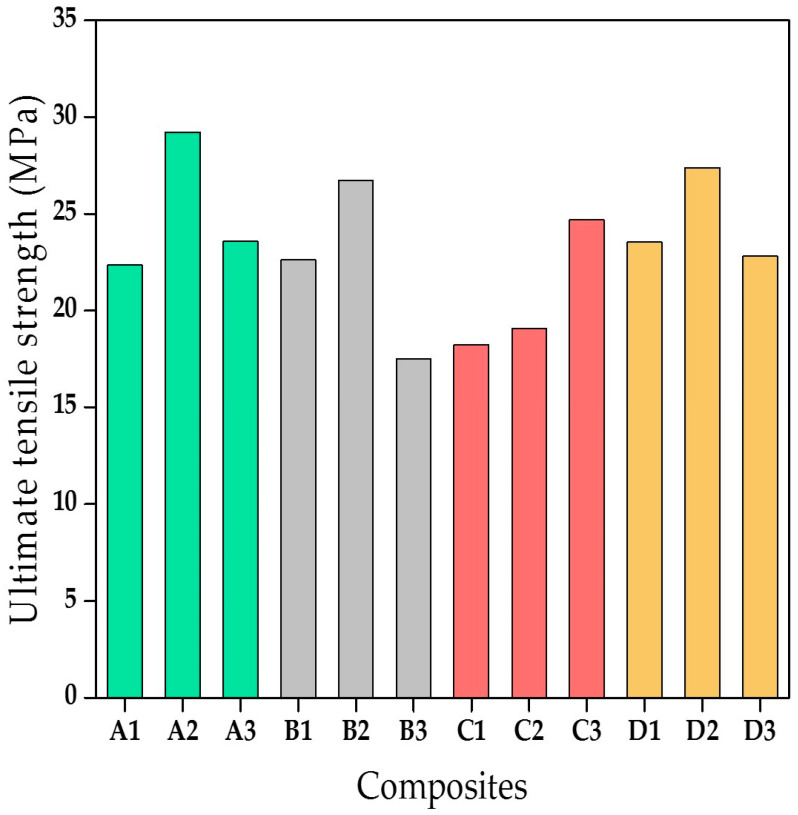
Tensile behaviour of composites.

Figure 3 shows that the A2 composite had maximum tensile strength, whereas the lowest tensile strength was seen in the B3 composite. It can be concluded that the optimum fibre length is 10 mm for maximum tensile strength. There was a slight reduction in tensile strength of composites with smaller and larger fibre lengths. The tensile properties of the composites fabricated in this study showed better tensile properties compared to the bagasse fibre-reinforced polypropylene composites and bagasse fibre-reinforced cardanol polymer composites [27,28,29]. Though, it must be noted that both of these have different matrices. The chemical treatment of the fibres led to an improvement in tensile properties owing to the reason that after surface treatment, the wax layer is removed from the fibre surface. This waxy substance contributed to ineffective fibre-matrix bonding and inferior surface wet-out [27,28]. The NaOH treatment of fibres improved the stiffness, and hence it improved the tensile properties due to the better distribution of the tensile loading. 

**Figure 4:**
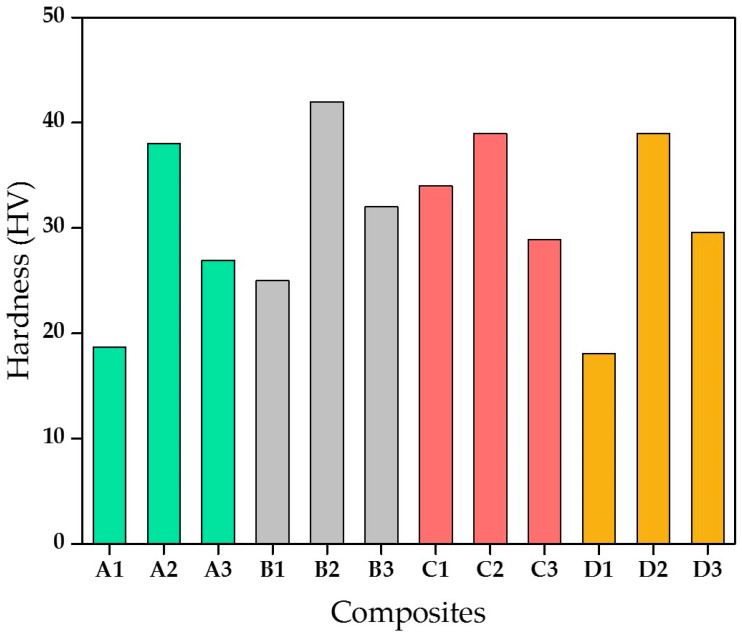
Hardness behaviour of composites.

Figure 4 shows that B2 composite had the maximum hardness, and D1 composite showed minimum hardness. It can be concluded that the optimum fibre length is 10 mm for higher hardness. The hardness of B and C composites was higher than A and D composites because B and D composites contain Cos 767 and CoJ 88 sugarcane bagasse fibre types, respectively, which are hard, whereas A and D composites contain Cos 8436 and Co 0239 sugarcane bagasse fibre types, respectively, which are soft (Table 1). Hence, the addition of a hard variety of fibre increased the hardness of the composites. 

**Figure 5:**
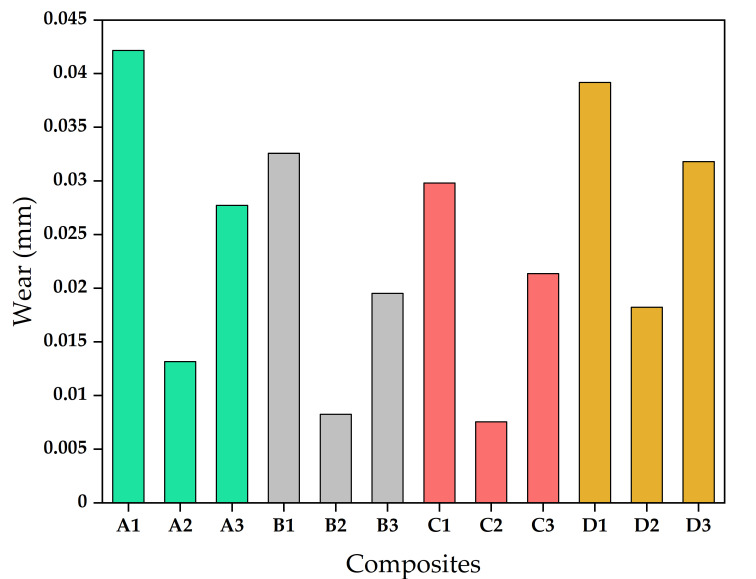
Wear behaviour of composites.

The wear was calculated in terms of length (mm) over a fixed period of time (10 min). In general, wear of the composite matrix leads to wear in the matrix, fibre wear, fibre fracture, and fibre–matrix interfacial debonding. Figure 5 represents the wear behaviour of different composites. B2 composite had the minimum wear, and A1 composite showed maximum wear. This is because the hardness plays an essential role in enhancing the wear resistance of the composites. The higher the hardness, the higher the wear resistance. It was observed that the optimum fibre length was 10 mm for the best wear properties.

**Figure 6:**
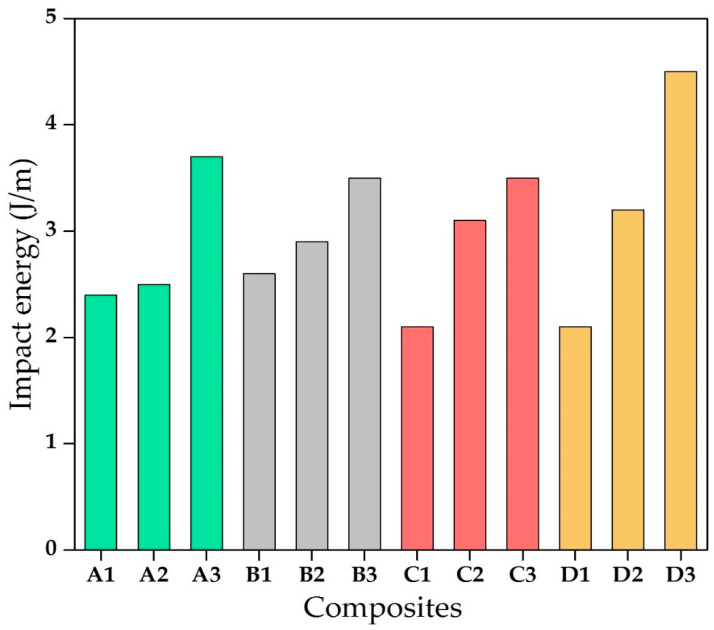
Impact behaviour of composites.

Figure 6 shows the ability of the different composites to resist impact loading. The fibre length plays a vital role in determining the impact strength of the composites and the dissipation of energy throughout the length of the composite. It was found that for higher fibre length, the impact carrying the load was high [30,31]. The impact energy was maximum for all varieties of bagasse fibre with 15mm fibre length. A similar trend is also reported for the bagasse fibre-reinforced composites [32].

### 3.3. Scanning Electron Microscope (SEM)

**Figure 7:**
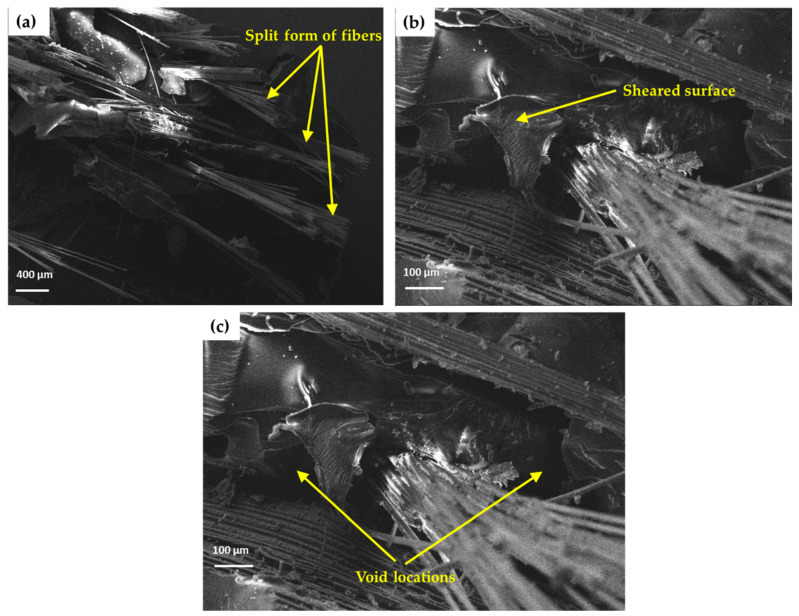
SEM photographs of (**a**) surface of the composite after tensile test, (**b**) sheared surface after wear test, and (**c**) void location in the composite among fibres.

A smooth surface of fibre can be ensured by chemical treatments, especially using NaOH treatment [26]. SEM images of the surface of tensile tested composites are shown in Figure 7a. It shows that after the treatment, the fibres split out from the composites packed together, known as fibrillation. The fibrillation also broke the untreated fibre bundles down into smaller ones by the dissolution of the hemicelluloses. Because of the fibrillation, the active surface area available for contact with the matrix increased. Hence, the interfacial bonding improved as reported by other researchers [33,34]. As shown in Figure 7b, the sheared surface after the wear test can be seen from the SEM photographs. An increment in the length of the fibre promoted the pull-out of the fibres from the matrix and debonding between the fibre and matrix took place. Figure 7c shows the void in the composite. The hand layup method of composite fabrication and other factors (not proper curing, impurities, etc.) caused the formation of voids in the composites.

### 3.4. Ranking of Materials Using the TOPSIS Method

For selecting the best possible alternatives from the available number of other options, the technique of order preference similarity to the ideal solution (TOPSIS) method is a powerful technique. This technique’s basic concept is to choose the best alternative from a set of multiple attributes or goals, which has the shortest distance from the ideal solution and the longest from the negative-ideal solution [35]. According to this method, among all the different options, the one which is closer to the positive ideal solution and farthest from the negative ideal solution is the best alternative. The main aim of the TOPSIS in the present study is to select the top-ranked composites based on their physical, wear, and mechanical characteristics and comparing it with all ranks in this set of all composites. The TOPSIS method was used to compare all the composites, and the ranking of the composites was carried out. The following Table 5, Table 6, Table 7 and Table 8 represent the decision matrix, normalisation matrix, weight normalised matrix, separation measure, relative closeness, and ranking, respectively.

The normalised decision matrix and the normalised value *n_ij_* was obtained using the following formula,

(1) $$n_{ij}\;=\;x_{ij}/\sqrt{\sum_{i=1}^{m}x_{ij}}$$

where *x* and *n* represent the element of decision and normalisation matrix corresponding to the value of *i* and *j*.

For the weight normalised matrix, the sum of weightage must be equal to one. We gave the following weightage for different properties.

(2) $$\sum_{1}^{m}=w_{j}=1_{j},$$

Here, we used the Shannon Entropy method for weight calculation and obtained weights for: Void fraction (%) = 0.15, Water absorption % = 0.13, Tensile strength = 0.13, Elongation % = 0.15, Hardness = 0.13, Impact energy = 0.13, and wear rate = 0.16.

The weighted normalized matrix elements were calculated by the following formula,

*r_ij_* = *w_j_* × *n_ij_* (3)

**Figure 8:**
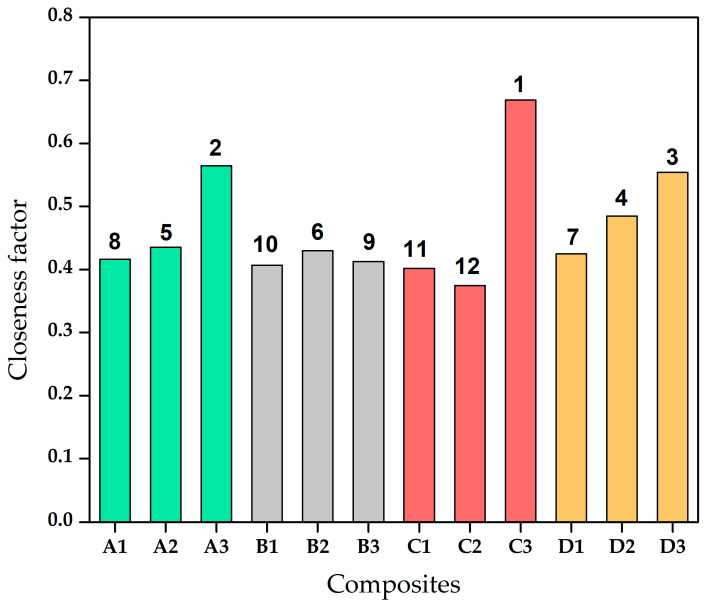
Ranking of composites based on physical, wear, and mechanical behaviour.

The ranking based on their physical, wear, and mechanical behaviour is shown in Figure 8 of the different composites that were analysed in this study. The method is devised to accommodate the positive effects of tensile and hardness and negative effect of wear and void fraction. The composite with rank 1—i.e., C3—was judged as the composite showing the best properties amongst the composites as per the TOPSIS method.

## 4. Conclusions

The experimental studies to estimate the physical and mechanical behaviour of bagasse fibre-reinforced epoxy composite was carried out. The following conclusions were drawn based on the present study:

- The minimum void fraction was obtained for composites reinforced with 5 mm length of all varieties of fibres because of the better compact composite formed with a small fibre length compared to 10 mm and 15 mm fibre length.
- The maximum ultimate tensile strength was obtained for the bagasse-reinforced natural composite of variety A and minimum tensile strength obtained for the C variety of bagasse-reinforced epoxy composite. Based on the tensile strength, the optimal length of fibre was 10 mm.
- The maximum elongation was obtained in the D composite reinforced with fibres of 10 mm in length and minimum in A composite reinforced with fibres of 5 mm in length. This is due to the ductile nature of 10 mm fibres compared to 5 mm, which showed a brittle nature.
- The maximum hardness was recorded for composite material B reinforced with a fibre length 10 mm and minimum for composite D reinforced with a fibre length of 5 mm. This occurred because B composites were fabricated from the hard plants of sugarcane and D from the soft plant of sugarcane.
- The maximum wear was obtained for A composite reinforced with a 5 mm fibre length and minimum for C composite reinforced with a 10 mm length. This is due to the lower hardness of A composite reinforced with a 5 mm fibre length and higher hardness of C composite reinforced with a 10 mm length
- The maximum impact energy was observed for the D composite reinforced with a fibre length of 15 mm because of the ductile nature of the fibre. Hence, it can absorb more energy before fracture.

## Figures and Tables

**Table 1:** Properties of different varieties of sugarcane bagasse fibre obtained from sugarcane.

Sugarcane Variety *	Physical Bending (Hard/Soft)	Cellulose Content (%) **	Hemicellulose Content (%) **	Lignin Content (%) **
Cos 8436	Soft	49.54	26.52	21.25
Cos 767	Hard	47.79	27.92	20.98
CoJ 88	Hard	48.45	27.39	21.11
Co 0239	Soft	47.86	28.25	19.81

* As per the nomenclature of the Indian Council of Agricultural Research (ICAR); ** by weight.

**Table 2:** Designation of composites.

Designation	Composition of the Composite
A1	Epoxy resin+ Cos 8436 sugarcane bagasse fibre of 5 mm length
A2	Epoxy resin+ Cos 8436 sugarcane bagasse fibre of 10 mm length
A3	Epoxy resin+ Cos 8436 sugarcane bagasse fibre of 15 mm length
B1	Epoxy resin+ Cos 767 sugarcane bagasse fibre of 5 mm length
B2	Epoxy resin+ Cos 767 sugarcane bagasse fibre of 10 mm length
B3	Epoxy resin+ Cos 767 sugarcane bagasse fibre of 15 mm length
C1	Epoxy resin+ CoJ 88 sugarcane bagasse fibre of 5 mm length
C2	Epoxy resin+ CoJ 88 sugarcane bagasse fibre of 10 mm length
C3	Epoxy resin+ CoJ 88 sugarcane bagasse fibre of 15 mm length
D1	Epoxy resin+ Co 0239 sugarcane bagasse fibre of 5 mm length
D2	Epoxy resin+ Co 0239 sugarcane bagasse fibre of 10 mm length
D3	Epoxy resin+ Co 0239 sugarcane bagasse fibre of 15 mm length

**Table 3:** Physical properties of the bagasse fibre composites.

Composites	Theoretical Density (g cm^−3^)	Experimental Density (g cm^−3^)	Volume Fraction of Voids (%)
A1	1.15	1.14 ± 0.04	1.02
A2	1.15	1.13 ± 0.02	1.943
A3	1.15	1.12 ± 0.03	2.62
B1	1.15	1.14 ± 0.01	0.97
B2	1.15	1.13 ± 0.07	1.835
B3	1.15	1.12 ± 0.02	2.811
C1	1.15	1.14 ± 0.03	0.95
C2	1.15	1.13 ± 0.02	1.77
C3	1.15	1.12 ± 0.04	2.655
D1	1.15	1.14 ± 0.03	1.075
D2	1.15	1.13 ± 0.04	1.90
D3	1.15	1.11 ± 0.05	3.306

**Table 4:** Mechanical properties of the bagasse composites.

Composites	Ultimate Tensile Strength (MPa)	Elongation (%)	Hardness (HV)	Impact Energy (J m^−1^)
A1	22.36 ± 2.7	0.56 ± 0.8	18.7 ± 2.5	2.4 ± 0.6
A2	29.23 ± 3.6	0.71 ± 0.5	38 ± 4.2	2.5 ± 0.2
A3	23.57 ± 2.7	1.05 ± 0.7	26.9 ± 3.6	3.7 ± 0.6
B1	22.63 ± 3.1	0.62 ± 0.3	25 ± 3.7	3.5 ± 0.4
B2	26.73 ± 2.4	1.21 ± 0.9	42 ± 5.3	2.9 ± 0.7
B3	17.49 ± 2.8	1.29 ± 0.2	32 ± 4.1	2.6 ± 0.6
C1	18.23 ± 1.5	1.03 ± 0.5	34 ± 3.6	3.5 ± 0.4
C2	19.07 ± 1.1	1.34 ± 0.4	39 ± 4.3	2.1 ± 0.3
C3	24.69 ± 2.1	0.95 ± 0.7	28.9 ± 5.1	3.1 ± 0.5
D1	23.54 ± 1.4	0.67 ± 0.5	18.1 ± 4.2	2.1 ± 0.2
D2	27.39 ± 3.3	2.19 ± 0.9	39 ± 3.4	3.2 ± 0.6
D3	22.82 ± 2.8	0.76 ± 0.1	29.6 ± 3.2	4.5 ± 0.9

**Table 5:** Decision matrix.

Samples	Void (%)	Water Absorption (%)	Tensile Strength (MPa)	Elongation (%)	Hardness (HV)	Impact Strength (J/m)	Wear Rate (μm)
A1	1.02	7.85	22.36	0.56	25	2.4	42.16
A2	1.943	8.56	29.23	1.21	39	2.5	13.17
A3	2.62	16.14	23.57	0.95	32	3.7	27.73
B1	0.97	9.97	22.63	0.71	38.1	2.6	32.58
B2	1.835	9.13	26.73	1.29	40.1	2.9	8.26
B3	2.811	7.12	17.49	0.67	36.2	3.5	19.53
C1	0.95	9.64	18.23	1.05	18.1	2.1	29.81
C2	1.77	9.02	24.6	1.03	39	3.1	7.56
C3	2.655	10.98	19.07	2.19	29.6	3.5	21.36
D1	1.075	10.17	23.54	0.62	32	2.1	39.17
D2	1.9	8.21	27.39	1.34	36	3.2	18.24
D3	3.306	10.35	22.82	0.76	28.9	4.5	31.81

**Table 6:** Normalisation matrix.

Composites	Void (%)	Water Absorption (%)	Tensile Strength	Elongation (%)	Hardness (HV)	Impact Strength	Wear
			(MPa)			(J/m)	(μm)
A1	0.1433	0.2265	0.2759	0.1445	0.2158	0.2245	0.4570
A2	0.2730	0.2469	0.3607	0.3123	0.3367	0.2338	0.1427
A3	0.3681	0.4656	0.2908	0.2452	0.2762	0.3461	0.3006
B1	0.1363	0.2876	0.2792	0.1832	0.3289	0.2432	0.3531
B2	0.2578	0.2634	0.3298	0.3329	0.3462	0.2713	0.0895
B3	0.3949	0.2054	0.2158	0.1729	0.3125	0.3274	0.2117
C1	0.1335	0.2781	0.2249	0.2710	0.1562	0.1964	0.3231
C2	0.2487	0.2602	0.3035	0.2658	0.3367	0.2900	0.0819
C3	0.3730	0.3167	0.2353	0.5652	0.2555	0.3274	0.2315
D1	0.1510	0.2934	0.2904	0.1600	0.2762	0.1964	0.4246
D2	0.2669	0.2368	0.3380	0.3458	0.3108	0.2993	0.1977
D3	0.4645	0.2986	0.2816	0.1961	0.2495	0.4209	0.3448

**Table 7:** Weight normalized matrix.

Samples	Void (%)	Water Absorption (%)	Tensile Strength (MPa)	Elongation (%)	Hardness (HV)	Impact Strength (J/m)	Wear Rate (μm)
A1	0.0215	0.0294	0.0359	0.0217	0.0281	0.0292	0.0731
A2	0.0409	0.0321	0.0469	0.0468	0.0438	0.0304	0.0228
A3	0.0552	0.0605	0.0378	0.0368	0.0359	0.0450	0.0481
B1	0.0204	0.0374	0.0363	0.0275	0.0428	0.0316	0.0565
B2	0.0387	0.0342	0.0429	0.0499	0.0450	0.0353	0.0143
B3	0.0592	0.0267	0.0281	0.0259	0.0406	0.0426	0.0339
C1	0.0200	0.0362	0.0292	0.0406	0.0203	0.0255	0.0517
C2	0.0373	0.0338	0.0395	0.0399	0.0438	0.0377	0.0131
C3	0.0559	0.0412	0.0306	0.0848	0.0332	0.0426	0.0370
D1	0.0227	0.0381	0.0378	0.0240	0.0359	0.0255	0.0679
D2	0.0400	0.0308	0.0439	0.0519	0.0404	0.0389	0.0316
D3	0.0697	0.0388	0.0366	0.0294	0.0324	0.0547	0.0552

**Table 8:** Separation measure, relative closeness, and ranking.

Composites	S^+^	S^−^	Closeness Factor
A1	0.091045	0.064919	0.416243
A2	0.078685	0.060697	0.43547
A3	0.058122	0.075338	0.564496
B1	0.084641	0.057976	0.406516
B2	0.081965	0.061836	0.430009
B3	0.082267	0.057828	0.412774
C1	0.084726	0.056849	0.401546
C2	0.087879	0.052604	0.374453
C3	0.048873	0.098607	0.668612
D1	0.086192	0.06369	0.424935
D2	0.06954	0.065467	0.484917
D3	0.063971	0.079484	0.554069
